# Capacitive and Infrared Gas Sensors for the Assessment of the Methane Number of LNG Fuels

**DOI:** 10.3390/s20123345

**Published:** 2020-06-12

**Authors:** Jörgen Sweelssen, Huib Blokland, Timo Rajamäki, Arjen Boersma

**Affiliations:** 1 TNO, HTC25, 5656AE Eindhoven, The Netherlands; jorgen.sweelssen@tno.nl (J.S.); huib.blokland@tno.nl (H.B.); 2 National Metrology Institute VTT MIKES, Tekniikantie 1, FI-02150 Espoo, Finland; Timo.rajamaki@vtt.fi

**Keywords:** energy transition, Methane Number, gas composition sensor, capacitive sensor array, interdigitated electrodes, responsive coatings, tunable filter infrared spectrometer, LNG, biogas

## Abstract

Liquid Natural Gas (LNG) is an energy source that is becoming more important in energy transition, as the world is facing lower the CO_2_ emissions and backup sources for wind and solar energy are needed. LNG is becoming a major player not only as fuel for power plants, but also in transport and mobility. However, the composition of LNG varies significantly between the various production locations around the world, and the layering of hydrocarbons with different molecular weights takes place even in LNG containers. This is especially critical for LNG engines, in which the ignition properties of the gas depend heavily on the fuel quality or Methane Number (MN) of the gas. For optimized engine operation and motor management, this fuel quality should be measured regularly, preferably online and by a small and low-cost sensor. This paper presents two sensor solutions for the assessment of the full gas composition. For both sensors, the standard deviation in the composition of the relevant hydrocarbons was low enough to calculate the Methane Number with an accuracy of approximately 1 MN unit. It was demonstrated that the electronic capacitive sensor was better suited to assess the higher hydrocarbons, whereas the infrared sensor showed higher selectivity for the lower hydrocarbons.

## 1. Introduction

The current energy transition from fossil fuels to renewable sources is a major worldwide challenge. One of the potential methods to facilitate this transition is the use of natural gas as a replacement of oil-based products in heating and transportation. This may facilitate a possible future transition to biogas and other renewable sources. Since natural gas is not produced at the locations of consumption, it is often compressed and cooled to Liquified Natural Gas (LNG) and transported by ships all over the world. LNG plays an increasingly important role as a transport fuel in gas and dual-fuel engines. However, it is well known that the composition of LNG depends heavily on the source and upgrading process. This composition has a major influence on the energy content and combustion properties of the fuel. Monitoring gas compositions and the associated Methane Number (MN) is critical for the optimal functioning of these engines when using LNG from different sources [1,2,3]. The methane number is a measure for the LNG quality or combustion performance of the gas.

Several sensor solutions have been proposed for the assessment of the composition and quality of natural gas, of which the majority are based on large laboratory equipment, such as Raman spectroscopy [4,5,6], gas chromatography [7], and infrared spectroscopy [8,9]. These laboratory analytical techniques generally perform very well with respect to selectivity and sensitivity but are too costly for ubiquitous gas quality monitoring for LNG engines. As a lower cost and smaller alternative, some new developments have been presented in the field of miniaturized micro electromechanical system (MEMS)-based natural gas sensors [10,11,12], which are based on the optical interaction of infrared light and chemical species present in the gas. One of the major challenges in the infrared-based detection of the components in (liquified) natural gas is the high chemical similarity of the hydrocarbon species. For this reason, an alternative gas sensor was developed with capacitive transducer technology, which enables the differentiation of various hydrocarbons in a natural gas mixture based on the combination of chemical interaction, critical temperature, boiling point, and size. This technology was presented in a previous paper for its relevance for the assessment of the composition of LNG, and was based on the absorption of gas molecules in porous absorbing coatings applied to a capacitive sensing platform [13,14,15]. In this paper, more detailed results were presented and a correlation was made with the Methane Number. A series of 16 reference gas mixtures (having high concentrations of n- and iso-butane, and n- and iso-pentane) were assessed with the new capacitive sensor array (CSA). The results, i.e., selectivity and sensitivity, were compared to a MEMS benchmark technology based on tunable filter infrared spectroscopy (TFIR) [16,17].

The previous study [15] showed that the selectivity of the sensor array was very good for higher hydrocarbons larger than propane but showed some significant cross-sensitivity for ethane and propane. In the present study, we modified some of the coatings used to reduce the cross-sensitivity and optimized the data processing in order to improve the selectivity of the lower hydrocarbons. In addition to the data from the coated chips, we also included a few combinations of coated chips (e.g., product or ratio of two chips). In this way, the temperature influence could be reduced and the responses to some gasses could be amplified. In this paper, we also calculated the MN of the various gas mixtures and assessed the optimal way of calculating the MN. We used the NPL (National Physics Laboratory, UK) algorithm [18] as a benchmark to calculate the MN from the composition.

## 2. Materials and Methods

### 2.1. Capacitive Sensor Array—Technology

**Figure 1:**
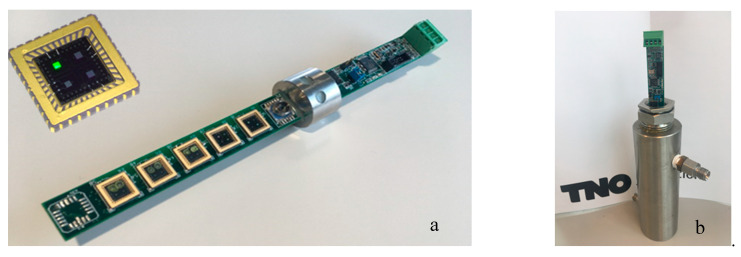
Sensor array Printed Circuit Board (PCB) including five coated sensing chips, having 10 functional electrodes (**a**). Sensor array in gas cell for exposure to gas mixtures (**b**).

The development and construction of the capacitive sensor array has already been discussed in detail in the previous paper [15]. In short, a series of capacitive sensor chips, consisting of aluminum interdigitated electrodes on a silicon substrate, was coated with the responsive coatings. The sensing chips were bonded to a Printed Circuit Board (PCB). The coatings absorbed the gasses in the LNG gas mixtures, which resulted in a change in the dielectric constant of the coating. This change was measured by the interdigitated electrode using AD7746 Capacitance-to-Digital converter chips (Analog Devices, Norwood, MA, USA), connected to the PCB. A microprocessor was applied to the PCB to read the digital signals from the AD chips to preprocess the temperature, pressure, and sensor chip data into a readable table and transmit the data via a USB connection to an external PC or laptop. The sensor is shown in Figure 1.

In the previous study [15], a series of eight coatings was synthesized, which gave a reasonable sensitivity and selectivity of the measured gasses over a large concentration range. We concluded that the cross-sensitivity of ethane and propane was high, and tried to mitigate this by the implementation of several alternative coatings. The list of coatings used is shown in Table 1.

Three coating formulations listed in Table 1 have been changed with respect to the previous study: Two MOF coatings and one Z26 coating, having no polymeric binder, and HAD, which is a soluble fluoropolymer Poly(tetrafluoroethylene-co- 2,2,4-trifluoro-5-trifluoromethoxy-1,3-dioxole) (Hyflon AD 40 HS, Merck, Darmstadt, Germany).

In addition to the values of the capacitances, a temperature and pressure reading was included in the data file. The temperature chip had a resolution of 0.1 °C, and the pressure sensor of 1 mbar. The microprocessor can send the data to the external computer with a frequency of ca 0.1 Hz.

### 2.2. Capacitive Sensor Array—Exposure Experiments

The sensor array that was manufactured, having ten coated electrodes, one temperature sensor, and one pressure sensor was exposed to various gas mixtures containing methane, ethane, propane, n-butane, iso-butane, n-pentane, iso-pentane, and nitrogen. A series of 20 different gas mixtures was used to calibrate the sensor array. The final validation was done with a series of 16 gas mixtures that were prepared at the Technical University of Braunschweig (TUBS) and calibrated at Physikalisch-Technische Bundesanstalt (PTB) [19]. This series was used for the comparison of the capacitive sensor and the Tunable Filter Infrared sensor. The pressure was set at 1.2 bar(a), and the temperature was not regulated, but only changed a few degrees during the experiment (~26 °C to 27 °C) as a result of fluctuating laboratory temperature and heating of the sensor electronics. The temperature difference between the calibration and validation experiments was within 1 °C. The composition of the gas mixtures was regulated by flow controllers (Bronkhorst High Tech, Ruurlo, Netherlands) and validated using a gas chromatograph (Compact GC4.0, Global Analyzer Solutions, Breda, Netherlands). The exposure time of the sensor to the gas mixtures was three hours for each mixture. This long period was chosen because some of the sensor chip respond relatively slow to changes in gas composition. The most important reason is the presence of contamination in the gas tubes in the gas mixing station. The calibration mixtures are compiled from the separate gasses, and when very low amounts of, e.g., butane or pentane are needed, the flow through the tubes is very low. This means that it can take a long time before all gasses are mixed and fed to the sensor in the required composition. In addition, when starting up the experiment or a flow in a tube, some water that has migrated into the system during hours of nonoperation needs to be removed first, before a reliable measurement can be done. The validation gas mixtures do not need to be compiled from the separate gasses, so exposure to the sensor can be shorter. Nevertheless, an exposure time of three hours was used as well for the validation.

The sensor array was first calibrated using a selection of the gas mixtures, and subsequently validated using the set of 16 mixtures that was supplied by TUBS/PTB [19]. The process and results of both the calibration and validation tests of a first series of experiments was discussed in the previous paper [15]. In this paper, we mainly focused only the results of the validation tests on the 16 standard gas mixtures from TUBS/PTB.

The 16 standard gas mixtures that were used for the comparison between the two sensing solutions are listed in Table 2. The values in the last columns are the Methane Numbers calculated from the composition using the NPL algorithm (MN(NPL)) and determined by TUBS/PTB using the engines test (MN(TUBS)).

The data processing has also been explained previously using simple multivariate matrix calculations. First a calibration matrix was generated using the test results of the calibration experiments. Then, this calibration matrix was used to calculate the composition and methane numbers from the chip data. In this paper, the processing was slightly different from the previous paper [15]. Instead of using only the ten sensor chips, in the current paper, we also used combinations of two chips: One additional signal for the product of two chips (Ch_n_ × Ch_m_), and one additional signal for the ratio of two chips (Ch_n_/Ch_m_). The extra signal of the product of two chips amplifies some of the signals for specific gasses, while the extra signal of the ratio of two chips reduces some of the responses that may cause cross-sensitivity and also reduces temperature effects. Since the data processing was multivariate linear regression using only linear dependencies on chip responses, combinations of chip responses were not included. So, by adding these two combined chip responses, we obtained twelve unique chip signals for the calculation of eight gas concentrations (CH_4_, C_2_H_6_, C_3_H_8_, n-C_4_H_10_, iso-C_4_H_10_, n-C_5_H_12_, iso-C_5_H_12_, N_2_). These two additional signals (Ch_n_ × Ch_m_) and (Ch_n_/Ch_m_) were added to the response matrix in Equation (1). The chip numbers *n* and *m* are different for each gas and are listed in Table 3. This means that the calibration procedure for each gas is different (using different combinations of signals). For the validation test, the same combinations of signals must be used. The temperature and pressure were not incorporated in the calibration and validation, since they were kept constant during all tests at 26.4 ± 0.4 °C and 1.2 ± 0.01 bar(a), respectively. When temperature and pressure fluctuate more, these parameters must be also included in the chip responses of Equation (1):

(1) $$\left[\begin{array}{c} Ch_{1}\\ \vdots \\ \begin{array}{c} Ch_{10}\\ Ch_{n}\times Ch_{m}\\ Ch_{n}/Ch_{m} \end{array} \end{array}\right]=\left[\begin{array}{c} \alpha_{1}^{1}\\ \vdots \\ \alpha_{1}^{12} \end{array}\;\;\;\begin{array}{c} \cdots \\ \ddots \\ \cdots \end{array}\;\;\;\begin{array}{c} \alpha_{8}^{1}\\ \vdots \\ \alpha_{1}^{12} \end{array}\;\;\;\right]\left[\begin{array}{c} \begin{array}{c} Gas_{1}\\ \vdots \end{array}\\ \begin{array}{c} Gas_{8} \end{array} \end{array}\right]$$

The gasses are listed in Table 3. This can be simplified to:

(2) [Ch]=[M][C]

[*M*] is the response matrix for all chips. For the calculation of the gas concentrations from the chip responses, the relation has to be rewritten into:

(3) $$\left[C\right]={\left[{\left[M\right]}^{T}\left[M\right]\right]}^{-1}{\left[M\right]}^{T}\left[Ch\right]$$

So, after obtaining the calibration matrix [*M*] from the calibration experiments, Equation (3) can be used to calculate the gas concentrations from the chip responses. The accuracy of this calculation was assessed by comparing the calculated gas concentration to the setpoint gas concentration and calculate the standard deviation of the difference.

### 2.3. Methane Number

The Methane Number of an LNG fuel is a measure for the combustion performance, just like the Octane Number for petrol engines. Unfortunately, there is no analytical relationship between the fuel composition and the MN. This parameter must be measured experimentally using a well-calibrated engine. In theory, a fuel with a MN of 100 has similar combustion properties as 100% methane. A fuel with a lower MN (e.g., 90) behaves like a mixture of methane and hydrogen (i.e., 90% CH_4_ and 10% H_2_). By assessing the knocking behavior of methane/hydrogen mixtures and comparing these with actual fuels, the experimental MN is obtained.

However, for sake of simplicity, to enable the calculation of the MN from the composition and not only rely on experimental engine tests, much research has been conducted to calculate or approach the MN using analytical models, such as Anstallt für Verbrennungsmotoren (AVL), Motorenwerke Mannheim (MWM), National Physical Laboratory (NPL), and Wärtsilä (WMN) [20,21]. Unfortunately, all of these MN calculators give different results. In the current paper, we used the NPL algorithm as a benchmark to calculate the MN of the calibration and validation gasses, and the values for the calculated MN from the composition that were generated by the sensors [18].

In addition to the calculation of the MN, the actual values of the sixteen validation gasses were determined by TUBS/PTB by engine tests [19]. The values for the MN of these mixtures, as calculated using the NPL algorithm and as measured, are listed in Table 2. The standard deviation of the difference between the two values was 1.18, with a very large contribution to this uncertainty of MIX3 containing just methane (94.6%), propane (4.9%), and nitrogen (0.5%).

### 2.4. Tunable Filter Infrared Sensor—Technology

In a TFIR detection method, the infrared (IR) light is passed through a gas mixture and analyzed using a MEMS-based IR filter and low-cost detector. This technology combines the small footprint and low-cost features from MEMS products with the chemical accuracy of IR analytical techniques. A great benefit of these IR-based methods with respect to other chemical sensors is the fact that the sensor is not in contact with the target gas, and can operate relative remotely from the cold fuel or hot exhaust gasses. Therefore, it is also typical to apply TFIR sensors as independent extractive gas analyzers in the same way as, e.g., analyzers based on Fourier transform infrared (FTIR) spectroscopy. However, being a simpler technique with no moving parts, TFIR is potentially more robust and economic solution than FTIR in applications where moderate sensitivity in sub-percentage levels of gas concentration is adequate.

The TFIR sensor applied in this work has been explained in more detail by the authors of [15]. It was based on NIRONE NIR Spectral Sensor module at wavelength range 2.0–2.45 µm by Spectral Engines Ltd. The module was connected with NIR broadband light source and gas absorption cell using optical fibers. The optical path length of the sample cell was 400mm, the cell volume was approximately 200 mL, and the cell was temperature controlled. The gas sample cell output was open to ambient air and the actual measurements took place in ambient pressure. The Spectral Engines Sensor Control software was used for system control and collection of measured spectra.

### 2.5. Tunable Filter Infrared Sensor—Exposure Experiments

In all absorption spectrum measurements with the TFIR sensor sample, the cell temperature was set to a constant temperature of 45.0 °C. The sample gas was flowing continuously through the sample cell and the sample cell output was in ambient pressure. The typical gas flow rate was 1–2 L/min, and accordingly, gas exchange time for the gas cell and system response time was approximately 10 s. The background spectrum was measured using pure nitrogen when starting the measurements of the day, and the stability of the sensor was controlled by occasionally measuring nitrogen again. During a set measurement time of two seconds, a total of 100 spectra was collected internally by the control software and their average was calculated as an output and recorded. Furthermore, typically 10 or more consecutive series of spectra were recorded during a one-minute-maximum actual measurement time for each gas mixture in order to estimate standard deviation of the results. The average of these spectra was used as calculated measurement result for the measured gas mixture.

## 3. Results

The two analytical instruments were compared with respect to the sensitivity and selectivity for the concentrations of hydrocarbons in the 16 standard gas mixtures of methane, ethane, propane, n-butane, iso-butane, n-pentane, and iso-pentane.

### 3.1. Capacitive Gas Sensor Array: Calibration

First, a calibration experiment was done using a similar concentration range as the validation gasses of Table 2. These experiments revealed that each coated sensing chip had a different response to each of the gasses. For this reason, the combination of two chips (i.e., Ch_n_ × Ch_m_ and Ch_n_/Ch_m_) was different for each gas. These combinations were optimized for all gasses and are listed in Table 3. It appears that the three lower hydrocarbons were well presented by the same combinations of PIM, Z26, MOF, and TAF. Apparently, the responses of these three gasses were amplified by the PIM and Z26 coatings, and the response of the MOF coating was corrected with the TAF coating. It was seen that the MOF coating was very sensitive for the higher hydrocarbons. So, to make this coating relevant for the smaller hydrocarbons, a TAF correction appeared to be required, since TAF was sensitive for the smaller hydrocarbons as well. The higher hydrocarbons were measured preferably by the MOF coatings. Only the iso-butane gas showed a deviation from the other higher hydrocarbons. The experiments showed that the iso-alkanes were very difficult to measure. The interaction with the coatings was much lower than for the n-alkanes.

**Figure 2:**
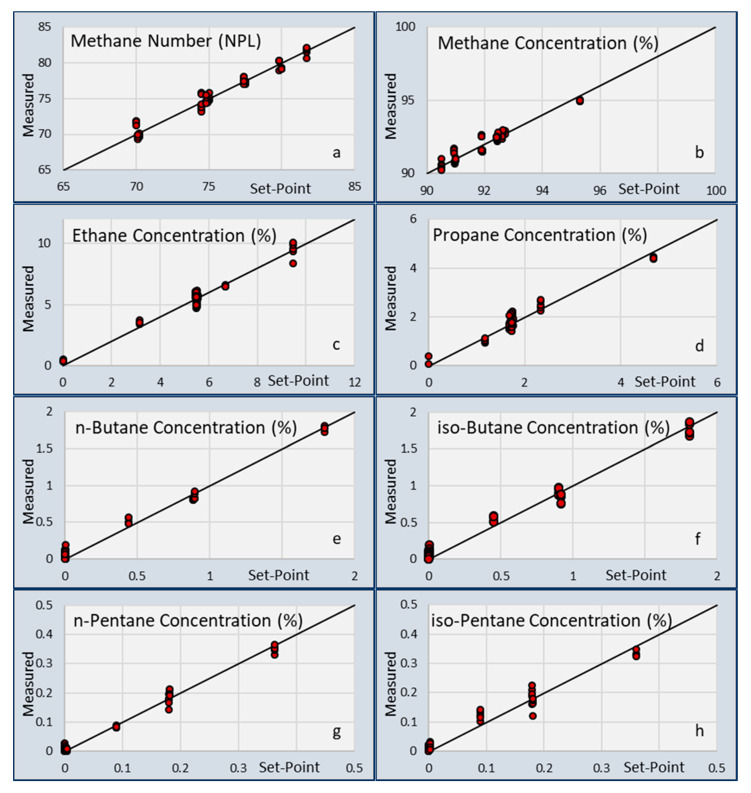
Calibration data for the capacitive gas sensor for the various gas parameters: (**a**) Methane number (NPL); (**b**) Methane; (**c**) Ethane; (**d**) Propane; (**e**) n-butane; (**f**) iso-butane; (**g**) n-pentane; (**h**) iso-pentane.

The calibration matrix [M] was obtained from these calibration experiments and used for recalculating the calibration concentrations and Methane Number. The calculated values of each of these gas parameters are plotted in Figure 2.

All the gasses in the calibration experiment can be well described by the linear matrix processing algorithms as presented in Equations (1)–(3). The standard deviation of the differences between setpoint gas concentration and measured concentrations are given in Table 4. When comparing the values from Figure 2 with the previous paper, a significant improvement is seen in the standard deviations. This indicates that the changes made on the new coating formulations and using two combinations of chip responses improved the correlations between the gas concentrations and the chip responses.

### 3.2. Capacitive Gas Sensor Array: Validation with 16 Standard Gas Mixtures

**Figure 3:**
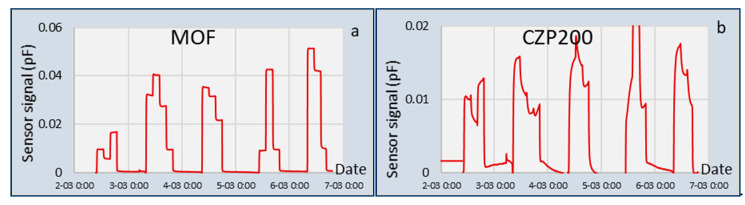
Example of two of the coated sensing chips exposed to the 15 gas mixtures: (**a**) Metal organic framework ZIF8 coating; (**b**) CZP200 zeolite coating.

The validation tests of the capacitive gas sensor were executed with 15 instead of 16 gasses, since one of the gas bottles (MIX5) was deflated during transport and storage. The temperature range of the validation test and pressure was almost equal to the calibration tests, so no correction for temperature and pressure were applied. The validation experiments were performed over five days, with each day having three or four subsequent tests. Between the testing days, the sensor was exposed to a nitrogen flow to keep moisture and other contaminants out of the system. The nitrogen signal before the first test was taken as the baseline, and all measurements were taken relative to the baseline signal. Two measured signals, corrected for the baseline, are shown in Figure 3. The sequence of exposure to the mixtures was: MIX1, MIX2, MIX3, MIX10, MIX12, MIX13, MIX14, MIX4, MIX6, MIX7, MIX8, MIX9, MIX11, MIX15, MIX16, MIX1. For all sensing chips, it was observed that switching from nitrogen to the gas mixtures showed an immediate increase in sensor signals (Figure 3). This means that all sensing chips responded very fast to changes in the gas composition. For some chips, the time needed to reach the final equilibrium value was long (e.g., chip CZP in Figure 3) due to slow gas absorption and/or release of water. Other chips responded very quickly (e.g., chip MOF in Figure 3). Once this chip showed a signal larger than 0, nitrogen was replaced by a gas mixture. At the end of every test series, when the gas mixture was replaced by nitrogen, the signal of this chip returned to 0 very quickly. This signal was then an indication of whether the sensor was exposed to nitrogen or a hydrocarbon gas mixture.

**Figure 4:**
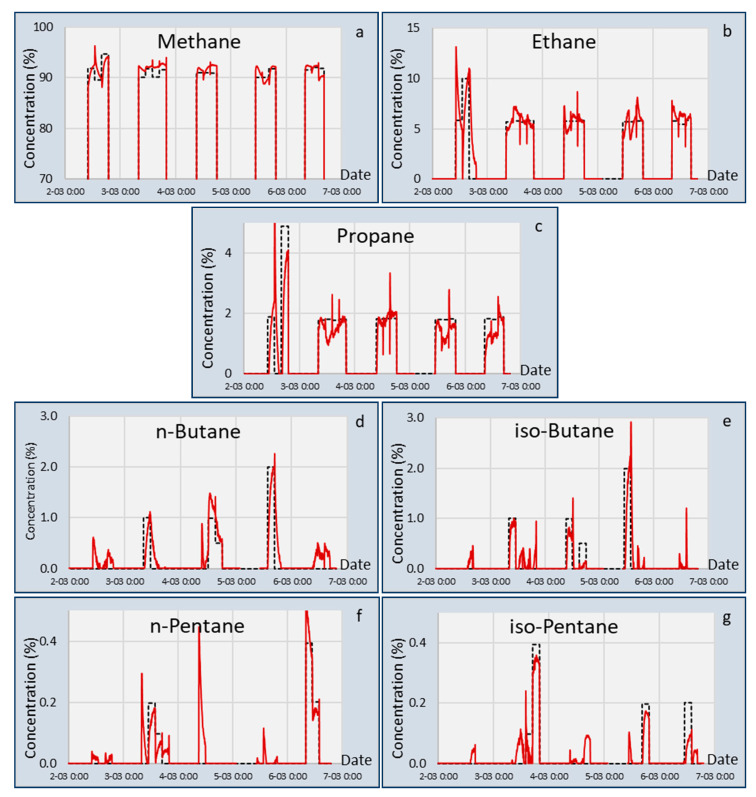
Calculated (red) and setpoint (black) gas concentrations in the validation gas mixtures: (**a**) Methane; (**b**) ethane; (**c**) propane; (**d**) n-butane; (**e**) iso-butane; (**f**) n-pentane; (**g**) iso-pentane.

The calibration matrix [M], obtained during the calibration experiments, was used to calculate the gas concentrations from the sensor data. The results of all seven gasses (excluding nitrogen) are shown in Figure 4.

Figure 4 shows that when switching from one mixture to the next, a spike in calculated concentration can be seen. The reason for this spike may have been a spike in pressure, water concentration, or temperature. The gas mixtures were delivered in gas bottles, and when connecting the bottles to the measurement system, water ingress can occur, resulting in the presence of a high concentration of water for a short period of time. In addition, it also takes time for one mixture to replace another. For this reason, the comparison between the calculated values and setpoint values was done for the last half hour of the three-hour exposure period. During this period, the 95% confidence interval and standard deviation between sensor measurement and experimental values were calculated for all gas components. These values are listed in Table 5 and Table 6.

The envisioned application of the sensor is its use in the assessment of the quality of LNG, in which the Methane Number is the most important parameter. The MN was calculated from the sensor data in three ways:

(1) FC(NPL): The methane number was calculated from the full composition using the NPL algorithm.
(2) FC(WMN): The methane number was calculated from the full composition using the Wärtsilä algorithm.
(3) MN(NPL): The methane numbers were calculated from the composition of the standard gas mixtures as given by TUBS/PTB and derived by GC using the NPL algorithm. Then, these values were used in the calibration procedure without using the composition of the gas mixtures.

These measured Methane Numbers were compared to the reference values. These values were also obtained in three ways:

(1) NPL: The Methane Number was calculated from the GC composition using the NPL algorithm.
(2) WMN: The Methane Number was calculated from the GC composition using the Wärtsilä algorithm.
(3) TUBS/PTB: The Spark Advance–Service Methane Number (SA-SMN) was obtained from direct measurements using a standard LNG engine.

**Figure 5:**
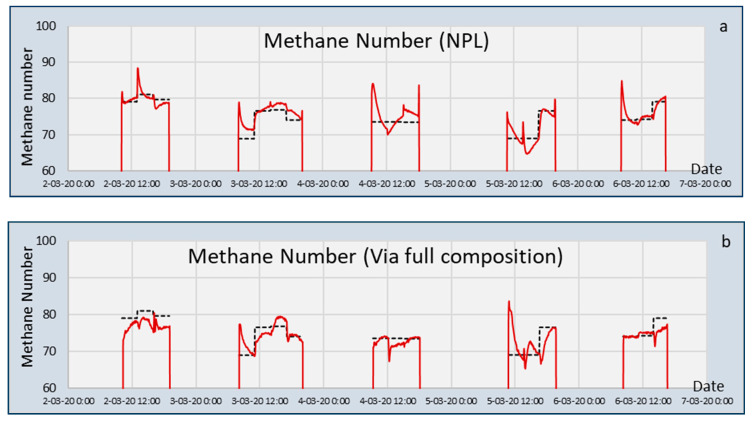
Calculated (red) and experimental (black) Methane Numbers of the validation gas mixtures, for (**a**) the MN(NPL) and (**b**) the full composition (NPL) approach.

The results for FC(NPL) and MN(NPL) for the capacitive sensor for calculating the Methane Number are shown in Figure 5. Some relevant differences between the two methods can be seen. The differences between the calculated and experimental values were slightly larger for the MNs calculated from the composition when looking at the last half hour of measurements. The standard deviation of the differences between the calculated and experimental MNs are listed in Table 6.

When assessing the whole three-hour measurement, it appears that the MN calculated from the composition followed the setpoint values more accurately, except two mixtures on day 2 and day 4. These show a large overshoot in the beginning, which corresponds to the large spikes seen in Figure 4 for n- and iso-pentane. The overshoots for the directly calculated MN (MN(NPL)) were much larger, especially on day three and five. The overshoots in the calculated composition were partly compensated by the undershoots for other gasses, which resulted in a flattened MN curve. For the directly calculated (MN(NPL)) method, the overshoots in the sensor signals were directly translated into a peak value of the MN.

### 3.3. Tunable Filter Infrared Sensor: Calibration

The TFIR sensor was calibrated separately for all studied hydrocarbons. The applied concentration ranges covered concentrations in typical LNG mixtures of different origin. Calibration was made using a suitable number of mixtures with different concentration: Methane 0–100 vol-% (13 mixtures), ethane 0–30 vol-% (7), propane 0–10 vol-% (5), n-butane 0–3 vol-% (5), iso-butane 0–3 vol-% (5), n-pentane 0–0.6 vol-% (3) iso-pentane 0–0.6 vol-% (3), and n-hexane 0–0.5 vol-% (3). Certified standard gas cylinders of these hydrocarbons by AGA (Finland) were used, and calibration gas mixtures with different concentrations were generated using calibrated mass flow controllers. Nitrogen was used as a balance gas for methane, and methane was used for other hydrocarbons. From the calibration spectra for different hydrocarbons an analysis algorithm was constructed applying the classical least squares optimization method. The same analysis algorithm and calibration spectra were used in previous work [15] for sensor testing and validation.

### 3.4. Tunable Filter Infrared Sensor: Validation with the 16 Standard Gas Mixtures

**Figure 6:**
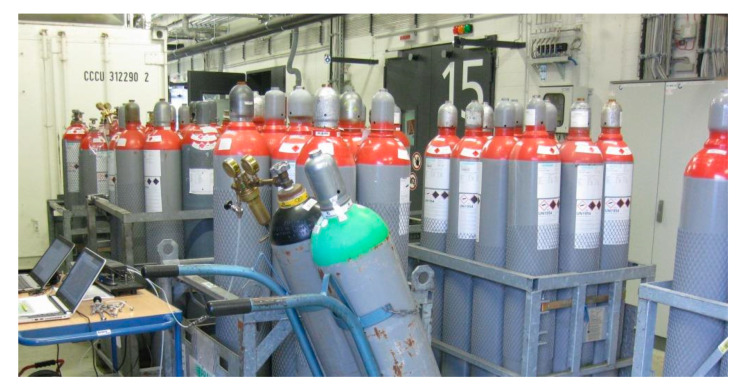
TFIR measurements of test gas cylinders took place in laboratory entry hall at TUBS (Braunschweig, Germany, details in [19]).

All 16 gas mixtures listed in Table 2 were measured with the TFIR sensor. The experiment took place at TUBS (Germany) using the original test gas cylinders. The working place for the measurements is shown in Figure 6. The large door of the laboratory entry hall where the measurements took place was occasionally opened to outdoor air, and on a hot summer day, the ambient temperature of the space increased by more than 5 °C during the day. However, due to the temperature control of the sample cell, the sensor output did not show a drifting behavior when the background level was occasionally tested with pure nitrogen. As a whole, it only took two hours to measure the spectra of gas mixtures from all 16 cylinders due to the fast response time and good stability of the TFIR sensor. A total of 64 spectra for all mixtures were recorded with a two-second measurement time for a single spectrum. To measure the background spectrum before starting the actual measurements, nitrogen was used to flush the sample cell.

**Figure 7:**
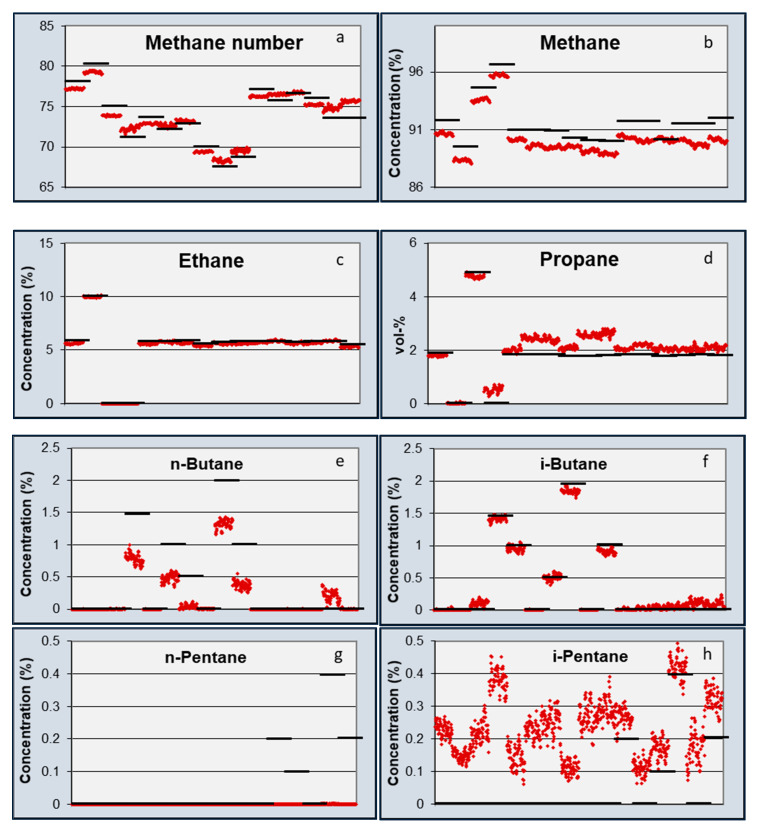
Measured (red) and actual (black) Methane Numbers and hydrocarbon concentrations (vol%) of the validation gas mixtures. (**a**) Methane number; (**b**) methane (**c**) ethane; (**d**) propane; (**e**) n-butane; (**f**) iso-butane; (**g**) n-pentane; (**h**) iso-pentane.

Measured and actual concentrations and Methane Numbers based on calculated concentrations for all 16 mixtures are shown in Figure 7. The standard deviation of the differences between test cylinder gas concentration as obtained by GC measurements at PTB and measured concentration from these measurements in 16 mixtures is given in Table 6. The results for concentrations were most stable and accurate in the case of ethane. Furthermore, methane, propane, and iso-butane were well analyzed and separated from each other. However, for n-butane, the results were significantly more scattered, and the results for iso-pentane were poor and hardy, as no n-pentane was detected from the gas mixtures. This was due to the similarity of the spectroscopic signatures of these molecules. The analysis algorithm tended to overestimate propane and n-butane concentrations when longer chain hydrocarbons were present in the gas mixtures. However, this cross-sensitivity was balanced in the calculated Methane Number values. From the results for the Methane Numbers of different mixtures, shown in Table 7, it is evident that the aforementioned inaccuracy in analyzing concentrations of single hydrocarbon molecules did not prevent determination of an accurate value for the Methane Number of the gas composition. In Table 7, Methane Numbers are furthermore calculated using the algorithm developed by Wärtsilä (WMN [20,21]). The average difference between the Methane Numbers calculated from measured compositions by TFIR and GC, determined with corresponding MN-algorithms for all 16 mixtures, was 0.9 MN units in the case of Wärtsilä Methane Numbers and 1.3 MN units in case of Methane Numbers calculated using the NPL algorithm. This corresponds well with the average error in Methane Numbers calculated using the Wärtsilä and NPL algorithms with respect to the Methane Number determined directly from engine tests made by TUBS (SA-SMN). In both cases, the value was 0.7 MN units.

### 3.5. Statistics

**Figure 8:**
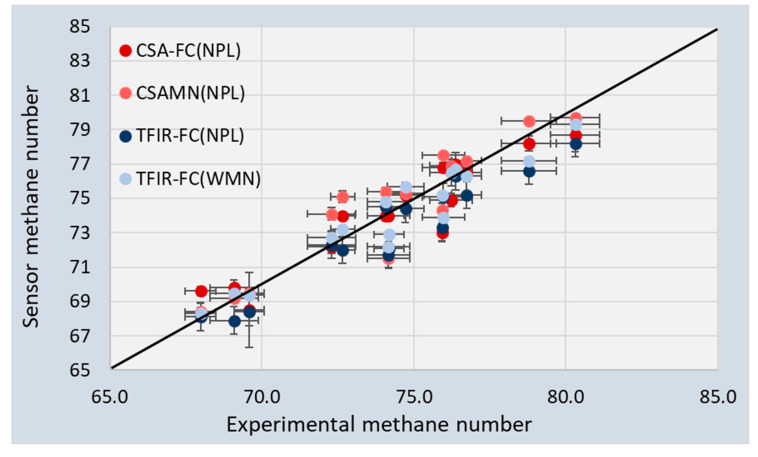
Sensor Methane Numbers plotted versus the experimental Methane Numbers from the TUBS/PTB engine tests (SA-SMN).

An important result of the sensor developments and validations presented in this paper is an estimation of the difference between the sensor measurements and experimental values for the Methane Number. Several uncertainties were introduced in all the measurements and calculations, starting with the uncertainties of the gas chromatograph, which was used to measure the compositions of both the calibration and validation mixtures. The sensors themselves also introduced uncertainties with respect to measured capacitance or optical absorption. For simplicity, the accuracy of the measurements of the concentrations and Methane Number was estimated using a 95% confidence interval by assuming a normal distribution of errors. The standard deviation for each gas (σ) was calculated for the measurements of Figure 4, Figure 5 and Figure 7 and using 2σ, the 95% CI was obtained. This 95% CI interval is shown in Table 5 for the individual gasses obtained by the two sensors and plotted in Figure 8 for the Methane Numbers.

The accuracy of the composition and Methane Number measurement is calculated by assessing the standard deviation between the measured values and the experimental values. These standard deviations are listed in Table 6 and Table 8 for the composition, and Methane Numbers.

## 4. Discussion

In Section 3, the results of the LNG composition and Methane Number measurements are presented using the two sensors: The capacitive sensor array (CSA) and tunable filter infrared sensor (TFIR). The 95% confidence intervals of the experiments of Figure 4, Figure 5 and Figure 7 are listed in Table 5 for the measured gasses and Methane Number. It is apparent that both sensors showed different features, but both calculated the Methane Number with a good 95% CI below 0.5 units, although the stability of the TFIR signal was better than the CSA signal. For the individual gasses, it was observed that the CSA 95% CI for methane was better and for ethane was poorer than the TFIR values. Furthermore, those for propane and butane were larger and for pentane were smaller than FTIR. This is an indication that the TFIR is more accurate for ethane, but can detect the pentanes only with a large error. This is also reflected in Table 6. From this table, it becomes clear that the TFIR sensor is more sensitive for the smaller hydrocarbons, and the CSA shows a higher accuracy for the larger hydrocarbons. Notably, ethane was still difficult to measure with the CSA, and gave a very accurate value using the TFIR. The reason for this difference may be found in the difference in sensing mechanism. The TFIR measured all gas components by the infrared assessment of the presence of chemical bonds in the mixtures. Every chemical bond has a proportional contribution to the total signal. So, low concentrations of components give a small contribution and generate a high uncertainty in the concentrations. The CSA is based on the absorption of gas components in responsive coatings. Since the absorption depends on the interaction between the gas and the coating chemistry, the absorption of one gas can be significantly higher than another gas. This is the case for the higher hydrocarbons that are preferably absorbed by most of the coatings. Another difference can be seen between the absorption of iso- and n-alkanes in the coatings. The signals of the n-alkanes were much higher than the iso-alkanes, which is represented in the higher accuracy of the n-alkanes in Table 6.

For the application of the two sensors for LNG composition measurements, some additional differences can be observed. The response times measured for the CSA were higher than for the TFIR. This was partly due to the experimental setup, in which the gas mixtures required some time to replace the previous mixture in the exposure chamber and remove all the water that was introduced by the switching of the gasses. However, the slower absorption rate of some gasses into the coatings also worsens the response times. When composition changes are small and slow, which typically is the case for an LNG burning engine using fuel from a large tank, it is expected that the CSA can follow these changes nicely, although the TFIR will be a faster sensor in all cases.

Second, once calibrated, the CSA can operate for a long period of time without additional calibration or baseline correction [13]. The validation experiments of the CSA lasted for five days and started two weeks after the calibration. A nitrogen baseline was measured just before the first exposure to MIX1 and was used for the whole series of experiments. The TFIR used a new nitrogen baseline in the beginning of the measurement day. This is possible in laboratory conditions but will be a challenge with in-line field implementation. Accordingly, high stability of the system construction is a benefit.

Furthermore, pressure and temperature corrections need to be included for both sensors. In the current paper, pressure and temperature were kept relatively constant and TFIR gas sample cell was temperature controlled, which contributed to the very good results. In practice, it has been shown for the CSA that pressure and temperature compensation are possible, provided the calibration also includes these temperature and pressure ranges [13]. The pressure compensation of TFIR sensor results is possible as well.

The Methane Numbers have been measured and calculated using several approaches. All of these values are listed in Table 7. It is clear that every approach resulted in a different value. There were also some differences between the calculated values using the two algorithms and the experimental values obtained by TUBS/PTB. The standard deviation of the differences between the experiments and the algorithms was around 1 MN unit. This shows that every way of obtaining the Methane Number has its own uncertainties. The values for the spark-advanced service Methane Number (SA-SMN) were taken from [19]. The 95% confidence intervals obtained for these experiments were in the range between 0.2 MN and 0.9 MN units, which is larger than the range of 0.25 MN to 0.5 MN units that was found for the CSA and TFIR sensors.

Figure 8 compares the sensor MNs with the experimental values (SA-SMN). The sensor data is not equally distributed above and below the black line. Furthermore, some of the sensor datapoints show a large deviation from the experimental value for all approaches. The 95% CI is shown in Figure 8 for both the sensor data and experimental data.

It was found that the 95% CI for the Methane Numbers for both sensors was 0.25–0.50 vol%. Figure 8 shows that the 95% CI for the experimental values was much larger: Between 0.2 and 0.9 vol% [19].

An overview of the maximum and average deviation is listed in Table 8. The table shows that the maximum differences between the calculated and the experimental Methane Numbers were similar for all approaches, at around 3 MN. Figure 8 and Table 8 show that the TFIR generally underestimated the MN with respect to the experimental values. On average, both CSA results had a standard deviation of 1.2–1.3. The TFIR results were slightly better when the WMN algorithm was used. A reason for this may be found in the slightly better correlation between WMN algorithm and SA-SMN. Furthermore, the results of the NPL and WMN algorithms using the GC data also showed a standard deviation of 1.1–1.2. This all indicates that the accuracy of both sensors is similar and can be compared very well with mathematical solutions. For the assessment of the Methane Number, the two sensors can be used equally well as a gas chromatograph.

The comparison of the presented sensors with alternative sensor solutions for the detection of the gas composition in a fuel or gas line is difficult, since there are not many examples published that enable the measurement of Methane Numbers. Many examples were presented in the literature for the measurement of methane concentrations (e.g., [22,23]). However, for the measurement and monitoring of the Methane Number or Wobbe Index, the detection of methane is not sufficient. A full composition of the fuel gasses is needed. Most full gas composition monitoring systems are based on GC or Raman, and they perform better than the capacitive sensor or the TFIR sensor with respect to selectivity (typical 0.1–0.3 vol-% accuracy), but this comes with a much higher price. Other composition monitoring solutions are comprised of a combination of various physical sensors (thermal conductivity, speed of sound, density, infrared sensor [24,25]) presented an accuracy of ~1% in the calculated concentrations or Wobbe Index. The accuracy in the results of such combined physical sensor approaches decreases upon the increase of the number of components in the gas. The authors of [25] showed that the accuracy decreased from 1% for binary mixtures to 3% for ternary mixtures. The authors of [24] presented an accuracy of 1% in the Wobbe Index, but no composition or Methane Number was measured or calculated. The FTIR solution by the authors of [19] only calculated the Methane Number with a comparable accuracy as found in the present paper (~1.5 MN units) but required a large laboratory device.

## Figures and Tables

**Table 1:** Coating formulations that were applied to the interdigitated electrodes. PTAF = soluble polytetrafluoroethylene, PIM = polymer of intrinsic micro porosity, PIFB = polyimide having fluorinated building blocks, MOF = metal organic framework, C200/C800/Z26 = zeolite, HAD = soluble copolymer.

Channel	Name	Polymer Matrix	Additive
1	PTAF	PTFE AF1600	–
2	PIFB	Fluorinated polyimide	–
3	PIM	PIM-1	–
4	MOFP	PIM-1	30 wt% ZIF-8
5	C200	PIM-1	50 wt% NH4CZP200
6	C800	PIM-1	50 wt% NH4CZP800
7&8	MOF (2×)	-	ZIF-8
9	HAD	Hyflon AD	-
10	Z26	-	H-ZSM5_26

**Table 2:** Composition (in vol%) of the 16 standard gas mixtures that were used for the sensor assessment, as supplied by TUBS/PTB [19].

MIX	CH_4_	C_2_H_6_	C_3_H_8_	n-C_4_H_10_	i-C_4_H_10_	n-C_5_H_12_	i-C_5_H_12_	N_2_	MN (NPL)	MN (TUBS)
MIX1	91.76	5.85	1.884	0	0	0	0	0.50	79.1	79.0
MIX2	89.49	10.02	0.00	0	0	0	0	0.49	81.1	80.5
MIX3	94.60	0	4.900	0	0	0	0	0.50	79.7	76.2
MIX4	96.60	0	0	1.455	1.455	0	0	0.49	74.1	74.4
MIX5	91.92	5.77	1.817	0	0.994	0	0	0.49	73.6	74.4
MIX6	90.91	5.77	1.829	0.993	0	0	0	0.50	73.6	72.5
MIX7	90.83	5.83	1.831	0.504	0.505	0	0	0.50	73.5	72.9
MIX8	90.00	5.72	1.801	0	1.990	0	0	0.49	69.1	69.8
MIX9	90.00	5.71	1.794	2.000	0	0	0	0.49	69.1	68.2
MIX10	90.02	5.70	1.781	1.004	1.005	0	0	0.49	69.0	69.3
MIX11	91.70	5.79	1.820	0	0	0	0.199	0.50	76.6	77.0
MIX12	91.69	5.80	1.810	0	0	0.198	0	0.50	76.6	76.5
MIX13	90.09	5.68	1.775	0	0	0.098	0.098	0.50	76.9	76.6
MIX14	91.49	5.81	1.804	0	0	0	0.396	0.50	74.1	76.2
MIX15	91.48	5.79	1.830	0	0	0.394	0.000	0.50	74.1	74.3
MIX16	91.96	5.45	1.800	0	0	0.202	0.203	0.38	74.3	74.9

**Table 3:** Combination of chip responses used in the calibration of each of the gasses.

Gas nr	Gas	Ch_n_ × Ch_m_	Ch_n_/Ch_m_
1	CH_4_	PIM × Z26	MOF/TAF
2	C_2_H_6_	PIM × Z26	MOF/TAF
3	C_3_H_8_	PIM × Z26	MOF/TAF
4	n-C_4_H_10_	MOF × MOF	TAF/C200
5	iso-C_4_H_10_	HAD × HAD	Z26/TAF
6	n-C_5_H_12_	MOF × MOF	TAF/C200
7	iso-C_5_H_12_	MOF × MOF	C800/HAD
8	N_2_	-	-

**Table 4:** Standard deviations of the calibration experiments compared to those obtained in the previous paper on the first prototype of the capacitive LNG sensor from [15].

Gas	Calibration This Paper	Calibration [15]
CH_4_	0.32	0.50
C_2_H_6_	0.38	0.78
C_3_H_8_	0.23	0.39
n-C_4_H_10_	0.09	0.18
iso-C_4_H_10_	0.08	0.14
n-C_5_H_12_	0.012	0.03
iso-C_5_H_12_	0.022	0.03

**Table 5:** 95% confidence intervals for the calculated gas concentrations and Methane Number of the two sensors (CSA and FTIR) calculated from the results of Figure 4, Figure 5 and Figure 7.

Gas	CSA (vol%)	TFIR (vol%)
CH_4_	0.14	0.25
C_2_H_6_	0.29	0.10
C_3_H_8_	0.13	0.13
n-C_4_H_10_	0.059	0.054
iso-C_4_H_10_	0.060	0.049
n-C_5_H_12_	0.009	0.059
iso-C_5_H_12_	0.007	-
MN	0.49	0.25

**Table 6:** Standard deviations of the differences between measured gas concentrations by gas chromatograph and the calculated gas concentrations in the 16 mixtures as obtained by the capacitive sensor array and TFIR sensor.

Gas	CSA (vol%)	TFIR Sensor (vol%)
CH_4_	0.58	1.23
C_2_H_6_	0.62	0.13
C_3_H_8_	0.34	0.44
n-C_4_H_10_	0.15	0.34
iso-C_4_H_10_	0.22	0.07
n-C_5_H_12_	0.035	0.12
iso-C_5_H_12_	0.056	0.20

**Table 7:** Methane Numbers, calculated from the CSA and TFIR sensor compared with the reference values as calculated using the NPL and Wärtsilä (WMN) algorithm and measured by the engine tests at TUBS/PTB (SA-SMN).

MIX	Capacitive Sensor Array		Tunable Filter Infrared Sensor		Calculated from GC Composition		Experimental Engine Results
	FC(NPL)	MN(NPL)	FC(NPL)	FC(WMN)	NPL	WMN	SA-SMN
MIX1	78.2	79.5	76.6	77.2	79.1	78.1	78.8
MIX2	78.7	79.7	78.2	79.3	81.1	80.3	80.3
MIX3	76.8	77.5	75.1	73.9	79.7	75.0	76.0
MIX4	74.0	71.5	71.7	72.2	74.1	71.1	74.2
MIX5	-	-	72.1	72.9	73.6	73.6	74.2
MIX6	72.2	74.1	72.3	72.7	73.6	72.1	72.3
MIX7	74.0	75.1	72.0	73.2	73.5	72.8	72.7
MIX8	68.5	69.5	68.4	69.4	69.1	70.0	69.6
MIX9	69.6	68.4	68.1	68.3	69.1	67.5	68.0
MIX10	70.0	70.5	67.9	69.5	69.0	68.7	69.1
MIX11	76.5	77.2	75.2	76.3	76.6	77.1	76.7
MIX12	74.9	76.9	76.5	76.5	76.6	75.7	76.2
MIX13	77.0	76.4	76.3	76.7	76.9	76.6	76.4
MIX14	73.0	74.3	73.3	75.2	74.1	76.0	75.9
MIX15	74.0	75.4	74.5	74.8	74.1	73.6	74.1
MIX16	76.9	75.8	74.4	75.7	74.3	73.6	74.7

**Table 8:** Maximum and average difference between the sensor Methane Numbers (CSA, TFIR) and the algorithm Methane Numbers (NPL, WMN) relative to the experimental Methane Numbers (SA-SMN).

Parameter	CSA FC(NPL)	CSA MN(NPL)	TFIR FC(NPL)	TFIR FC(WMN)	NPL	WMN
Maximum	2.9 (MIX14)	2.7 (MIX4)	2.6 (MIX14)	2.0 (MIX3)	3.5 (MIX3)	3.3 (MIX4)
Average	−0.17	0.33	−1.03	−0.33	0.11	−0.68
Standard deviation	1.2	1.3	1.5	1.0	1.2	1.1
